# The Biology and Evolution of the Three Psychological Tendencies to Anthropomorphize Biology and Evolution

**DOI:** 10.3389/fpsyg.2018.01839

**Published:** 2018-10-01

**Authors:** Marco Antonio Correa Varella

**Affiliations:** Department of Experimental Psychology, Institute of Psychology, University of São Paulo, São Paulo, Brazil

**Keywords:** anthropomorphism, teleology, mentalizing, intentional stance, theory of mind, natural selection, education, misunderstandings

## Abstract

At the core of anthropomorphism lies a false positive cognitive bias to over-attribute the pattern of the human body and/or mind. Anthropomorphism is independently discussed in various disciplines, is presumed to have deep biological roots, but its cognitive bases are rarely explored in an integrative way. Conversely, I present an inclusive, multifaceted interdisciplinary approach to refine the psychological bases of mental anthropomorphism. I have integrated 13 conceptual dissections of folk finalistic reasoning into four psychological inference systems (physical, design, basic-goal, and belief stances); the latter three are truly teleological and thus prone to anthropomorphisms. I then have integrated the genetic, neural, cognitive, psychiatric, developmental, comparative and evolutionary/adaptive empirical evidence that converges to support the nature of the distinct stances. The over-reactive calibration of the three teleological systems prone to anthropomorphisms is framed as an evolved design feature to avoid harmful ancestral contexts. Nowadays, these stances easily engage with scientific reasoning about bio-evolutionary matters with both negative and positive consequences. Design, basic-goal, and belief stances benefit biology by providing cognitive foundations, expressing a high-powered explanatory system, promoting functional generalization, fostering new research questions and discoveries, enabling metaphorical/analogical thinking and explaining didactically with brevity. Hence, it is neither feasible nor advantageous to completely eliminate teleology from biology. Instead, we should engage with the eight classes of problems in bio-philosophy and bio-education that relate to the three stances: types of anthropomorphism, variety of misunderstandings, misleading appeal, legitimacy controversy, gateway to mysticism, total prohibition and its backfire effect. Recognizing the distinction among design, basic-goal, and belief stances helps to elucidate much of the logic underlying these issues, so that it enables a much more detailed taxonomy of anthropomorphisms, and organizes the various misunderstandings about evolution by natural selection. It also offers a solid psychological grounding for anchoring definitions and terminology. This tripartite framework also shed some light on how to better deal with the over-reactive stances in bio-education, by organizing previous pedagogical strategies and by suggesting new possibilities to be tested. Therefore, this framework constitutes a promising approach to advance the debate regarding the psychological underpinnings of anthropomorphisms and to further support regulating and clarifying teleology and anthropomorphism in biology.

## Introduction

**FIGURE 1:**
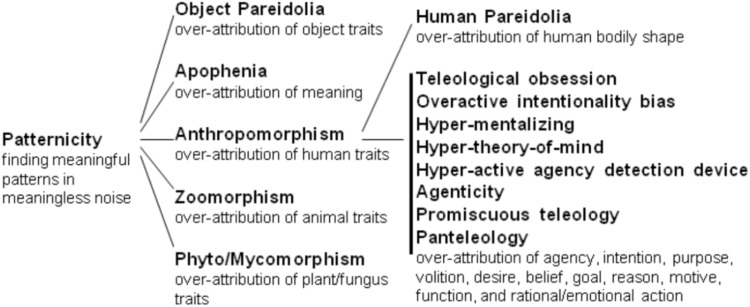
Possible organization of the conceptual relationship among the many types of over-attribution tendencies within patternicity. In general, those tendencies occur respectively when we see specific objects, meanings, human forms/minds, non-human animal forms/minds, plant forms, or fungus forms where there is only vague similarity or none.

The search for pertinent pattern is the world is ubiquitous among animals, is one of the main brain tasks and is crucial for survival and reproduction. However, it leads to the occurrence of false positives, known as patternicity: the general tendency to find meaningful/familiar patterns in meaningless noise or suggestive cluster (Shermer, 2008). Patternicity can be visual, auditory, tactile, olfactory, gustatory or purely psychological. It varies from enabling normal analogical reasoning, in which the process of schema transfer from a familiar domain is intentionally used to clarify a problem in another domain (Wong, 1993), to pathological cases of hallucinations (Waters and Fernyhough, 2017). Patternicity is an umbrella term encompassing different kinds of over-attribution (**Figure 1**). Among related phenomena there is *anthropomorphism*: finding the pattern of human body and/or intentional mind where there is only vague similarity, suggestive resemblance, noise or nothing.

Is anthropomorphism just a mistake or a potent adapted bias? Is it something we should suppress or exercise with precision? This review is focused on integrating the biological foundations and psychological scope underpinning the tendency toward anthropomorphism, particularly the over-interpret of mentality where there is none. I firstly present its widespread status throughout several disciplines and highlight that the authors often presume a deep biological root for the tendency toward mental anthropomorphism. Do we really have an evolved built-in propensity to anthropomorphize? If so, how many psychological systems are engaged along the process? I then organize several conceptual dissections converging toward a tripartite division of the main cognitive faculties leading to mental anthropomorphism. Afterward, I present a cross-disciplinary summary of evidence offering a biological foundation of the three distinct mental capacities, pointing to adaptive values.

In the second half of this review, I show that the same psychological capacities prone to anthropomorphize are activated within biological sciences. Do they hinder or aid to advance the biological reasoning? After presenting its positive consequences, I show how the comprehensive and tripartite view of the psychological scope underlying mental anthropomorphism can illuminate their negative consequences to biology, such as organizing the misunderstandings about natural selection. Should we avoid the mistaken explanations or come up with ways to used them in favor of a more intuitive and accurate understanding? At the end, I present some pedagogical strategies known to be effective for teaching evolution and new ones to be tested based on this framework. I hope to advance the philosophical and educational debate concerning mental anthropomorphism by providing interdisciplinary evidence about the foundation and tripartite nature of the cognitive tendencies prone to anthropomorphize biology and natural selection. The same way humans were able to tame the destructive nature of fire to get light, heat, cooked food, locomotion, up to fire juggling, it seems feasible and productive to train the anthropomorphic tendencies for the best once uncovering its inner properties.

### The Widespread Status of Anthropomorphism

Anthropomorphism is widespread in both of its branches. Human pareidolia occurs when we see humanoid figures/faces in clouds, landscapes, rocks, or other objects (Guthrie, 1993). Neuroscientific evidence shows that women are more prone than men to see faces where there are none (Proverbio and Galli, 2016). The tendency for perceiving and preferring faces in face-like stimuli is present in newborn human infants (Johnson et al., 1991; Simion et al., 2001) and in juvenile monkeys raised without exposure to real faces (Sugita, 2008). Thus, familiarity and deep phylogenetically inherited knowledge about how humans (primates) look and behave play a role (Eibl-Eibesfeldt, 1989).

Anthropomorphic pareidolia lies in the evolutionary roots of human representational artistic propensity (Morriss-Kay, 2010; Varella et al., 2011b; Bednarik, 2016). Varella et al. (2011a,b, 2012) defended an evolutionary trajectory of paleoart aesthetics that started with a preexisting propensity to perceive/prefer patterns of anthropomorphs, zoomorphs, social scenarios and skillfulness that were later co-opted to recognize/appreciate paleoart visual content. Later this cooptation was expanded particularly through sexual selection into artistic instincts. Indeed, the earliest paleoaesthetics evidence points to the capacity of pre-sapiens, possibly *Homo heidelbergensis*, to detect anthropomorphic properties of objects and to improve it, such as in the case of the proto-figurine from Tan-Tan (300k - 500k BP) and Berekhat Ram (250k - 280k BP) (Bednarik, 2003; Morriss-Kay, 2010). The oldest case of face pareidolia dates from 3 million years ago, before the genus Homo. A 5cm dark red jasperite pebble, known as Makapansgat cobble, has natural makings in the appearance of a face and was found in a cave of Australopithecine. There is no intentional modification to the pebble which originated at least 32 km away from the cave. Thus, it was carried to the cave, possibly because of the hominid’s capacity toward anthropomorphic facial pareidolia being activated by the suggestive form of the pebble (Bednarik, 1998; Morriss-Kay, 2010). Similarly, today the *Chinsekikan* Museum (The Hall of Curious Rocks) in Japan houses over 1,700 rocks that naturally resemble human faces, including Elvis Presley (Nace, 2016).

Conversely, the mental branch of anthropomorphism is fascinating given its psychological nature. It is the false positive bias of over-attributing agency, intention, purpose, volition, desire, belief, goal, reason, motive, function, and rational/emotional action where there is only vague suggestive similarity or none. A ‘rock’ example comes from the ‘sailing’ stones from Death Valley (California). These stones leave behind long parallel, almost linear, track marks that are suggestive of self-propelled movement and rational choice for the shortest route toward an targeted place. Actually, Norris et al. (2014) discovered that melting thin ice sheets underneath the stones and light winds generate the apparently purposeful movement.

Many authors reserve the use of the term anthropomorphism only for its mental branch (e.g., Fisher, 1996; Reiss, 2017). The concept is so widely relevant that authors from various perspectives independently call it different names (**Figure 1**). Bacon (1878) referred to “**idols of the tribe**” when he stressed that human nature is such that it sees final causes (goals, reasons) everywhere even though they belong ‘only’ to human nature and not to the nature of the universe *per se*. Richard Dawkins referred to it as “**purpose colored spectacles**” during his BBC special “The big question: Why are we here?” in 2006^1^. Within cognitive ethology, it is called “**Intuitive anthropomorphic bias**” (e.g., Dacey, 2017), and it helps to clarify how to better interpret non-human behavior. In the intersection between cognitive science and robotics, it is called “**Anthropomorphic projection**” (e.g., Airenti, 2015), where it helps to explore possible meaningful interactions between people and artificial intelligence agents. Within cognitive psychology, it is called “**Teleological obsession**” (e.g., Csibra and Gergely, 2007) or “**Overactive intentionality bias**” (e.g., Rosset, 2008), where it helps to better understand why people pervasively presume intentional action in all behaviors. Within psychiatry, it is known as “**Hyper-mentalizing**” (e.g., Badcock, 2004) or “**Hyper-theory-of-mind**” (e.g., Clemmensen et al., 2014), where it helps understanding episodes of paranoia, persecutory thinking and related delusions in schizophrenic patients. Evolutionary theories of religiosity call it “**Hyper-active agency detection**” (e.g., Guthrie, 1993; Barrett, 2000; Boyer, 2001), where it helps to understand animism and the origins of the widespread belief in supernatural beings/deities. Within critical thinking and skepticism, it is known as “**Agenticity**” (e.g., Shermer, 2011), where it helps understanding the prevalent interest in conspiracies, paranormal events and supernatural/intelligent beings, such as ghosts and aliens. Within bioscience education, it is known as “**Promiscuous teleology**” (e.g., Kelemen, 2012), where it helps understanding why students often have a “function compulsion” of attributing intentionally designed use to everything. Finally, within bio-philosophy it is known as “**Panteleology**” (e.g., Mahner and Bunge, 1997), where it helps to distinguish theoretical conceptions attributing finality to all things in the cosmos, from those that attribute it only to some things (Hemiteleology). Importantly, those terms are not exact synonyms because they vary in the extension of the meaning. “**Teleological obsession**” and “**Overactive intentionality bias**” have the narrowest meaning, because they refer to anthropomorphizing of ‘only’ all human behavior (even involuntary ones), while “**Promiscuous teleology**” and “**Panteleology**” have the broadest meaning, because they refer to anthropomorphizing of everything in the cosmos.

### Mental Anthropomorphism as Built-In Default Bias

As outlined, the existence and importance of mental anthropomorphism is convergent and recognized across life domains. A crucial step for achieving this level of interest is the recognition that anthropomorphism is more than just a jargon or category mistake (Fisher, 1996). Rather, it is a result of a specific underlying cognitive bias that is somehow overly active (e.g., Broaddus, unpublished; Shermer, 2011; Airenti, 2015; Engvild, 2015; Dacey, 2017). Reiss (2017) argued that the notion that anthropomorphism is only a source of error that needs to be reconsidered. Fisher (1996) affirmed that the charge of anthropomorphism oversimplifies a complex issue. Dacey (2017) stated that anthropomorphism-as-an-error underestimates its complexity and that in order to better understand and control it, we must treat it as a cognitive bias. Following in the steps of Piaget, Chomsky, Tversky, and Kahneman, the idea is to take seriously the mistake: as a window to explore and uncover new facets of the human mind. This shift of focus can build a common base to further explore and to integrate the phenomenon.

**FIGURE 2:**
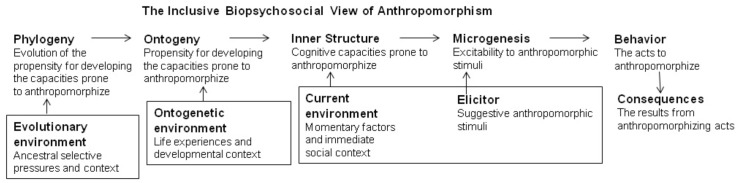
Inclusive bio-psychosocial integration of the factors related to anthropomorphizing in different time scales under different environmental influences; inspired by the way ethologists distinguish among behavior, its elicitor, its capacities, development and evolution (cf., Hinde, 1982; Hogan, 2017).

Further, there is general agreement that mental anthropomorphism is a “strong and early inclination” (Csibra and Gergely, 2007, p. 60), a “powerful bias” that “runs very deep,” “the default mode” (Gregory, 2009, p. 167), a “deep-seated tendency” (Rose and Schaffer, 2017, p. 243), that “feels natural” and “automatic,” an “innate disposition,” a “hard-wired tendency” (Broaddus, unpublished, p. 2, 4, 11), it is “simply built into us” (Kennedy, 1992, p. 28), “involuntary” and “pervading human thought and action” (Guthrie, 1993, p. vii–viii), and “at least as old as humankind” (Mahner and Bunge, 1997, p. 367). These descriptors convey the notion that mental anthropomorphism has impressive biopsychological roots. To further evaluate these assertions, it is important to distinguish among the objects triggering anthropomorphism, the anthropomorphic act, the capacities and its readiness, the propensity to develop the capacities and its evolution (**Figure 2**). Additionally, when focusing on “innate,” “natural,” and “hard-wired” one easily may forget about the importance of learning and of environment (cf., Dar-Nimrod and Heine, 2011; Heine, 2017). Thus, in **Figure 2**, I integrate the biopsychosocial influences in each time scale.

## Aims

Given its widespread status, growing convergent interest, and the presumed bio-psychological roots of mental anthropomorphism, I aim to dissect and to integrate the main points about the biology, cognition, development and evolution of psychological tendencies to over-attribute mentality, in order to build a comprehensive view. I then use this inclusive view to illuminate cases and issues of anthropomorphism in biology and evolution, as well as to help promote more effective strategies to deal with its positive and negative sides. This approach is aligned with evolutionary educational psychology (Geary, 2002) and with other attempts aimed at integrating different perspectives about mental anthropomorphism and its implications for understanding life (e.g., Whiten, 1991; Broaddus, unpublished; Galli and Meinardi, 2011; Shermer, 2011; Airenti, 2015; Engvild, 2015; Dacey, 2017; Dink and Rips, 2017). The main difference is that I propose a tripartite approach for the psychological scope of mental anthropomorphism.

## The Plurality of Teleological Reasoning Underlying Mental Anthropomorphism

The more we discover about anthropomorphism, the more the usual unitary view becomes an impediment. A pluralist approach puts things in perspective, contextualizes the problem, integrates disparate ideas, and fosters new hypotheses and conclusions. An important step toward a refined multifaceted view about mental anthropomorphism is to avoid an essentialist bias. Psychological essentialism is another highly accessible intuitive mode of thought that has five related components: stability, boundary intensification, within-category homogeneity, causes inherent in individuals, and existence of ideal categories (Gelman and Rhodes, 2012). Thus, in order to counteract the essentialist intrusive tendency, it is vital to think about teleological reasoning and its over-extended case in gradual terms, stressing flexibility, overlaps, heterogeneity and diversity, internal and external causes, imperfections, as well as considering the existence of versions in other animals (cf., Heine, 2017).

Although the overall mode of reasoning that configures mental anthropomorphism is commonly framed as teleological reasoning, i.e., thinking that generates a style of explanation dealing with goals, purposes, and reasons (e.g., Mahner and Bunge, 1997; Broaddus, unpublished; Engvild, 2015), it does not follow necessarily that its underlying biological, cognitive and evolutionary processes must be unitary. However, teleological reasoning “rests on poorly understood psychological primitives” (Schoemaker, 1991, p. 205), and many authors tend to assume its cognitive base stems only from folk psychology (e.g., Godfrey-Smith, 2009). A plural conceptualization of folk teleological tendencies toward mental anthropomorphism is found in philosophy (e.g., Dennett, 1989, 2017; Mahner and Bunge, 1997; Mayr, 2004); psychology (e.g., Rosset, 2008; Apperly and Butterfill, 2009; Schaafsma et al., 2015); development (e.g., Gergely and Csibra, 2003); and neuroscience (e.g., Saxe et al., 2004).

Therefore, in order to set the stage for integrating the factors related to mental anthropomorphism, **Table 1** organizes 13 ways in which underlying teleological cognition has been conceptually dissected into sub-domains by different authors with philosophical or psychological backgrounds. These sub-domains of teleological reasoning are referred to as specialized cognitive mechanisms or its products: ascriptions, stances, psychologies, representations, inference systems, languages, or modes of thought. Despite some inconsistencies usually stemming from different frameworks of authors, the clear pattern is the convergent and consistent division of teleological reasoning into specific sub-domains (**Table 1**). Most authors devise two or three sub-domains, but by analyzing all approaches together, a four sub-domain solution seems to be all-encompassing and stronger (cf., Boyer, 2001).

**Table 1:** Integration of 13 plural conceptualizations of the teleological reasoning according to which phenomena it is thought most suitable to apply.

Natural effects	Advantageous specialized use	Optimized self-interested patterned actions	Optimized self-interested reasoned inventive tactics	Intuitive focus
Inorganic/physical phenomena	Tools, body parts, social role	Prey, predators	Human conspecifics	Phenomena directed

*Endpoint- Attaining Systems*	*Designed Goal- Achieving Systems*	*Designed Goal-Pursuing Agent*	*Designed Goal-Intended Believing Agent*	Phenomena specified
• No design/proper function	• Artificial or natural design/proper function	• Natural or artificial design/proper function	• Natural or artificial design/proper function	
• No agent goal	• No agent goal	• Agent goal	• Agent goal	
• Nor belief	• Nor belief	• No false belief	• False belief
			Authors

–	Functional ascription	Goal-ascription	Intention-ascription	Beckner, 1969

Teleomatic language	Teleonomic language	Teleological language		Mayr, 1974, 2004
Physical stance	Design stance	Intentional stance		Dennett, 1989
–	–	Desire psychology	Belief-desire psychology	Wellman, 1991
Causal formulation	Non-anthropomorphic teleological reasoning		Anthropomorphic teleological reasoning	Tamir and Zohar, 1991

Physical mechanics mode of construal	Functional/teleological mode of construal	Folk psychology mode of construal		Keil, 1994

–	Teleonaturalism		Teleomentalism	Allen and Bekoff, 1995
–	–	Behavior-reading ability	Mind-reading ability	Whiten, 1996

Intuitive physics system	Structure-function system	Goal-detection system	Intuitive psychology system	Boyer, 2001

–	Functional stance	Teleological representation	Mentalistic representation	Gergely and Csibra, 2003/Csibra and Gergely, 2007

Mechanism, mode of cognition		Mentalism, mode of cognition		Badcock, 2004

Systemizing system		Intentionality detector	Theory of mind mechanism	Baron-Cohen, 2005

–	–	Low-level mindreading	High-level mindreading	Apperly, 2011

In general, the sub-domains are specialized to track different relevant phenomena, as two kinds of systems and two kinds of agents (**Table 1**). The **physical** stance tracks the natural inorganic systems without signs of design nor inorganic beneficiaries of possible effects. The **function/design** stance tracks naturally or artificially designed systems (i.e., parts of living beings, tools) presenting proper functions and having individuals and replicators (i.e., genes and memes) as beneficiaries of the achieved beneficial effects. The physical stance grasps incidental background lawful processes, while the function/design stance focuses on programmed mechanisms designed by selective processes (i.e., natural selection or learning/creating by trial and error). Here, the mind’s sub-domains distinguish between assigning ‘attained effects’ to the first sort of system as a part of our intuitive physics, and ‘beneficial function’ or ‘design’ to the second type, as part of the intuitive engineering, intuitive functional morphology, and intuitive social role of an individual within a group. One can also distinguish between two varieties of agents: (1) agents with the means to pursue important pre-set general goals with relative efficiency without much representing, conceiving, premeditating, or resetting the goals, *versus* (2) agents that on top of that also are able to represent, conceive, premeditate, even reset their goals, and learn how to find the most competent way of achieving them via intermediary goals. Here, the teleosub-domains track the distinction between basic desired-goals as part of intuitive behavioral analysis (e.g., of prey/predators) *versus* belief as part of our more elaborated intuitive psychology/ ethics.

Although far from exhaustive, **Table 1** offers a plausible starting point for considering the plurality of cognitive mechanisms generating thoughts about important recurrent aspects of our ancestral environment: Physical phenomena, tools/bodily parts/social role, prey/predators, and conspecifics. The last three of these cognitive mechanisms (design/functional stance, basic-goal stance, and belief stance) are genuinely teleological, and may produce acts of mental anthropomorphism. The common internal functioning among the three teleological stances is a presumption of rationality/optimization tiding together a predictive triangulation involving Desire/Need/ Want/Goal/Aim, Perceive/Belief/Know/Situational Constraints, and Intentional/Deliberated/Volitional Action (Dennett, 1989; Gergely and Csibra, 2003; Hudson et al., 2018). However, there could be other teleological cognitive mechanisms not yet properly described/integrated. One suggestion is the intuitive, broad sense of purpose in life (Bronk, 2013), which is studied within eudaimonic well-being, related to a meaningful/virtuous life.

This section encouraged a pluralistic conceptualization for teleological reasoning that avoids essentialist thinking and presented a tripartite cognitive subdivision (design, basic-goal, and belief stances) prone to mental anthropomorphism. In order to attest the reality of this plurality and their presumed bio-psychological roots, the next section will explore the main cross-disciplinary factors of cognitive underpinnings of mental anthropomorphism, mostly the belief stance. This focus is needed because this mode of thought, which enables us to explicitly attribute and to consider higher order mental states, including false-beliefs and deception, has been well-studied across many fields for many decades. It started as “naive psychology of action” (Heider, 1958), but it was also named theory-of-mind (ToM, Premack and Woodruff, 1978), intentional stance (Dennett, 1989), folk psychology (Wellman, 1991), mindreading (Whiten, 1991), mentalizing (Frith et al., 1991), and cognitive empathy (Zaki and Ochsner, 2016). The rare studies focusing on the other teleological stances will also be covered.

## Proximate and Distal Evolutionary Facets Underpinning Mental Anthropomorphisms

Here I highlight and integrate the main findings of each discipline about the sub-domains of folk teleology, mostly of the belief stance, and present evidence for their distinctions.

### Genetics

Twin studies have found modest to moderate heritabilities for tests of ToM, indicating some genetic variability underlying the individual variation in mentalizing and showing that environmental/cultural factors are responsible for the majority of the individual variation. Hughes and Cutting (1999) investigated 119 3-year-old twin pairs and found a 67% average estimate of heritability. The other 33% was explained by unique environment: the idiosyncratic child-specific factors non-shared within families. Hughes et al. (2005) found a 15% estimate of heritability for ToM in a sample of 1,116 5-years-old twin pairs. Ronald et al. (2006) assessed over 600 9-year-old twin pairs and found heritability of 12%, unique environment influencing 66% and shared environment influencing 22% of the variation. Melchers et al. (2016) found a 27% heritability for cognitive empathy in 742 twins and non-twin siblings.

In a meta-analysis, Warrier et al. (2017) investigated underlying genetics to ToM and its relation to other psychological traits and subcortical brain volumes. They performed genome-wide association in 88,056 participants, and additionally 1,497 twin participants. They confirmed a female advantage in mentalizing (Cohen’s *d* = 0.21) which may be partly due to different genetic architectures in men and women, interacting with post-natal social experience. They found that a locus in chromosome 3 (3p26.2) is associated with the ToM only in females. They found an average twin heritability of 28%, while the other two-thirds is explained by the non-shared environment. However, heritability was positively correlated between males and females, which indicates general genetic communalities. Genes related to higher capacity for ToM correlated with openness to experience, cognitive aptitude, educational attainment, and anorexia nervosa. Although not significant, the same genes for increased ToM correlated with bigger dorsal striatum, which consists of the caudate nucleus and the putamen. One of the genes within the locus in chromosome 3 is the Leucine Rich Neuronal 1, which is highly expressed in the striatum, related to social cognition.

### Neuroscience

Neuroscience has discovered specific brain areas related with mentalizing both cortical and sub-cortical. Gallagher and Frith’s (2003) review concluded that three cortical areas are consistently activated during tests of ToM: the superior temporal sulci, the temporal poles bilaterally, but principally the anterior paracingulate cortex. Abu-Akel and Shamay-Tsoory (2011) concluded that ToM primarily engages the dorsomedial prefrontal cortex, the dorsal anterior cingulate cortex and the dorsal striatum, and is dependent on the dopaminergic and serotonergic systems. Zaki and Ochsner (2012, 2016) concluded that mentalizing engages a specific system of midline and superior temporal structures (medial prefrontal cortex, temporoparietal, and superior temporal sulcus junctions), which are separate from empathic experience sharing. Overall, theory-of-mind’s cortical profile is stably active at rest (within the ‘default network’ indicating spontaneity/readiness), and is related to autobiographical memory, detection of biological motion, mental navigation, ‘self-projection’ into the future, past, counterfactuals and targets’ perspectives (Zaki and Ochsner, 2016). The sub-cortical portion, striatum, is related to social cognition and is activated by aversive/intense or novel/unexpected stimuli.

Lewis et al. (2014) investigated the neuroanatomy of subcomponents of eudaimonic wellbeing and found that ‘purpose in life’ is related to right insular cortex volume, and that there also was a marginally negative association with middle temporal gyrus volume. Thus, the neurocognition of ‘purpose in life’ seems to be different from mindreading. Reynaud et al. (2016) reviewed neuroscientific evidence on tool use, including planning and execution, which is related to the functional reasoning of design stance. They found brain regions, such as the left inferior parietal cortex, to be largely unrelated to those of ToM.

Importantly, Saxe et al. (2004) stated that neuroscience reinforces and elaborates upon the distinction between basic-goal and belief-goal cognitive systems by providing anatomical and functional evidence that domain-specific brain regions exist for representing belief contents, and that these regions are distinct from other regions engaged in reasoning about goals and actions. The temporoparietal junction, superior temporal sulcus, and medial prefrontal cortex show a strong activation for both true and false belief attributions. Conversely, brain regions involved in representing goal-directed action include the posterior superior temporal sulcus and Broca’s area. Similarly, Mar et al. (2007) found that social processing brain areas are especially tuned to realistic visual representations of conspecifics, because the related cortical areas are more active when mentalizing about live-action social agents than about cartoon agents. This suggests that basic-goal stance and belief stance are two distinct systems, rather than variations of a single system (Saxe et al., 2004).

### Cognition

Mentalizing is cognitively demanding and requires focus. It can be disrupted into an egocentric interpretation by the absence of time, effort, and attention. This indicates that mentalizing is initially processed with the assumption that the self shares states with targets and latter it requires an effortful correction of the assumption (Apperly and Butterfill, 2009; Zaki and Ochsner, 2016). Bradford et al. (2018) found for individuals from both Western and non-Western cultures that self-oriented belief-attribution was faster and more accurate than other-oriented belief-attribution.

Importantly, several authors agree with the psychological distinction between basic-goal and belief systems (Wellman, 1991; Boyer, 2001; Gergely and Csibra, 2003; Baron-Cohen, 2005; Apperly and Butterfill, 2009; Edwards and Low, 2017). Nevertheless, the two systems are connected (Apperly and Butterfill, 2009). Based on ontogenetic and neurocognitive evidence, Baron-Cohen (2005) proposed that ToM receives input from the shared-attention system, which in turn receives inputs from systems focused on detecting emotion, intentionality (i.e., basic-goal stance), and eye-direction. Similarly, Schaafsma et al. (2015) disentangled ToM into tracking of intentions and goals, moral reasoning, separation of knowledge and fact, understanding of causality, and emotion/gaze processing. Moreover, basic-goal and belief systems share similar mechanisms. Gergely and Csibra (2003) proposed a common denominator to represent actions by relating relevant aspects of reality (action, goal-state, and situational constraints) through the principle of rational/optimal action, which assumes that actions most efficiently realize goal-states (cf., Hudson et al., 2018).

Based on neuroanatomical and neurochemical evidence, Abu-Akel and Shamay-Tsoory (2011) proposed three levels of cognitive functionality of ToM: representation, attribution, and execution/application of mental states. The ability to represent ToM may be lost by damage to posterior brain regions, particularly the temporo-parietal junction. The ability to attribute mental states to self or others and to distinguish between them may malfunction after damage to the dorsal attentional systems that integrate the temporoparietal junction and anterior cingulate cortex regions via the dorsal lateral prefrontal cortex. The manner in which the individual applies mental states, toward hypo- or hyper-mentalizing, may malfunction after disruption to lateral prefrontal cortex structures, particularly related to increased dopamine or to neurochemical processes that modulate its functioning, such as the serotonin system (Abu-Akel and Shamay-Tsoory, 2011). The latter is directly related to the over-attribution nature of mental anthropomorphism and is intensely studied in psychiatry.

### Psychiatry

Abu-Akel and Shamay-Tsoory (2011) linked ToM impairment to over 20 psychopathologies ranging across psychiatric, genetic and neurological disorders. Different psychiatric conditions present selective impairment in mind-reading, while the rest of cognition remains normal. This dissociation particularly between hypo- and hyper-mentalizing, offers strong evidence for modularization of mind-reading. A typical hypo-mentalizing disorder is degrees of autism/Asperger’s spectrum, while schizophrenic individuals are diagnosed with hyper-mentalizing (Badcock, 2004; Brüne and Brüne-Cohrs, 2006; Crespi and Badcock, 2008; Abu-Akel and Shamay-Tsoory, 2011; Zaki and Ochsner, 2016). Thus, the execution/application component is calibrated along a continuum from low to high mental over-attribution. Moreover, Crespi and Badcock (2008) analyzed genetic, physiological, neurological, and psychological evidence as underpinnings of the psychotic-spectrum and proposed that maternally expressed genes promote hyper-mentalizing, and paternally expressed genes hypo-mentalizing.

Over-sensitivity to intention in schizophrenic individuals can take two forms: Positive, which underlies erotomania, or negative, which is much more common and relates to paranoia (Badcock, 2004). In schizophrenia, ToM deficits are repeatable, stable, heritable, have identified genetic markers, and distinctively disrupted neuro-functioning (Walter et al., 2011; Martin et al., 2014). Anthropomorphism in paranoid schizophrenia may result from either a mind-reading system that does not work properly or that is over-active (Abu-Akel and Shamay-Tsoory, 2011). Walter et al. (2009) found over-activity in the paracingulate cortex and the temporo-parietal junction to be associated with mental over-attribution in paranoid schizophrenics. Shermer (2011) concludes that patternicity may be associated with high levels of dopamine in the brain. He highlighted that increased dopamine is related to reward, pleasure, increased belief, pattern detection and false positives, and in higher doses triggers psychotic symptoms, such as hallucination and paranoia. Dopamine is also associated with enthusiasm and expectation (Shiota et al., 2017).

According to Baron-Cohen (2005), autistic children are able to represent the dyadic mental states of seeing and wanting (i.e., basic-goal stance) but show delays in shared attention and in understanding false belief (i.e., ToM). Atherton and Cross (2018) highlighted that ToM deficits in autistic individuals are ameliorated if the stimuli presented are cartoon or animal-like (i.e., basic-goal stance) rather than in human forms. Prothmann et al. (2009) found that autistic children (aged 11 years) interacted most frequently and for longest with a dog, followed by a person and then a toy. Furthermore, according to Badcock (2004) studies show that autistic children do not differ from others in their ability to understand the functions of an internal organ like the heart (i.e., design/functional stance). Moreover, autistic individuals have accentuated and precocious mechanical understanding and fascination with rule-based systems (i.e., physical stance) (Frith et al., 1991; Badcock, 2004; Baron-Cohen, 2005). Therefore, autism presents a case in which physical, design, and basic-goal stances are dissociated from the belief stance.

Similarly, Lombrozo et al. (2007) found that, compared to normal individuals, Alzheimer’s patients broadly accept and prefer teleofunctional explanations particularly for the existence of living organisms (trees, dogs), non-living natural entities (mountains, sun), and natural phenomena (rain, wind). However, a review of evidence on ToM in patients with neurodegenerative diseases concluded that there is a deficit of the cognitive ToM component in Alzheimer’s patients (Poletti et al., 2012). Therefore, this discrepancy provides further evidence that design and basic-goal stances are dissociated from belief stance.

### Development

In general, there is a well-defined, specific and universal ontogenetic route for understanding other agents. A meta-analysis on development of ToM using 178 studies found that false-belief performance showed a reliable developmental pattern across various countries and various task manipulations: Preschoolers went from below-chance to above-chance performance on false-belief tasks (Wellman et al., 2001). Beyond false-belief, Saxe et al. (2004) reviewed the literature and concluded that there is extensive evidence indicating that understanding other minds follows a characteristic developmental trajectory, beginning in the first 2 years of life with the early appearance of a system for reasoning about other’s goals, perceptions, and emotions, and, around 4 years of age, starts the maturity of another system for reasoning about other people’s beliefs. Similarly, Baron-Cohen (2005) placed the emergence of intentionality detection between 0 and 9 months and ToM at 4 years. Thus, very young children can attribute basic goals and desires much earlier than beliefs.

Ontogenetic evidence clearly supports distinct psychological mechanisms. Because brain regions associated with belief attribution are somewhat distinct from regions engaged with other people’s goals, the two stages of development established in the literature result from differential maturation of two distinct mechanisms, rather than from gradual improvement of a single mechanism (Saxe et al., 2004). Gergely and Csibra (2003) agreed with this distinction and further argued that even 1-year-old infants possess a naive theory of rational action that allows them to interpret/predict other agents’ goal-directed actions in a variety of different contexts using a non-mentalistic interpretational system. Csibra (2008) found evidence for goal attribution to inanimate agents in 6.5-month-old infants. Apperly and Butterfill (2009) reviewed evidence from development, cognitive sciences and comparative psychology and supported the existence of two agent-interpreting systems: An efficient but inflexible capacity for tracking basic belief-like states, that in humans persists in parallel with the later-developing, more flexible but more cognitively demanding ToM capacity. However, Onishi and Baillargeon (2005) and Buttelmann et al. (2009) used different tasks and found evidence for understanding false belief already in 15–18 year-olds.

Greif et al. (2006) found that children apply different logics to man-made artifacts *versus* animals: Children showed more curiosity about location and proper niche for animals but were more concerned with function and functioning for artifacts. Furthermore, children never asked what the animals were made for, which suggests that design stance and basic-goal stance are domain-specific separated mechanism. Keil (1994) found that second-graders preferred teleological explanations for biological kinds and mechanistic explanations for non-biological kinds. Concordantly, Kampourakis et al. (2012b) used open-ended questions and found that students provided teleological explanations for the features of organisms and artifacts but not for those of natural objects. Kampourakis et al. (2012a) argued that there is a conceptual shift in teleological thinking in which children up to 5 years show an unrestricted use of teleo-functional explanations, as found by Kelemen (2012) and Kelemen et al. (2013), but at later ages children use less teleofunctional explanations, mostly for parts of organisms and artifacts, and mostly for shape. However, future longitudinal research is needed to confirm this pattern (Kampourakis et al., 2012a).

### Ethology/Comparative Psychology

Evidence from our closest living relatives, the great apes, also supports the distinction between basic-goal and belief stances (Apperly and Butterfill, 2009). Call and Tomasello (2008) reviewed 30 years of comparative evidence and concluded that chimpanzees understand the goals and intentions of others, as well as the perception and knowledge of others. However, there was no evidence that chimpanzees understand false beliefs in terms of fully human-like belief psychology. Recently, Krupenye et al. (2016) and Buttelmann et al. (2017) used a modified task and demonstrated that great apes (chimpanzees, bonobos and orangutans) operate, at least on an implicit level, with an understanding of false beliefs, which lies already within the realm of the belief stance. Maybe in the future there will be evidence of belief stance in self-conscious animals. It is expected that we see a gradual instead of a sharp distinctions between humans and other apes, but until further replication one can conclude that the common ancestor of humans and chimpanzees 7.65 ± 1.01 million years ago (Pozzi et al., 2014) may have attributed basic-goals and desires to living agents much earlier than attribute beliefs. Moreover, reviewing evidence from 20 non-human species (mammals and birds), Emery and Clayton (2009) largely supported the distinction between basic-goal and belief stances, in concluding that non-human animals are excellent ethologists, but poor psychologists.

Wobber et al. (2014) did a cross-sectional and longitudinal study comparing physical and social cognition of 2- to 4-year-old human children and of chimpanzees (*Pan troglodytes*) and bonobos (*Pan paniscus*) in the same age range. They found that in physical cognition (space, causality, quantities), 2-year-old children and *Pan* apes performed comparably, but by 4 years of age children advanced and apes persisted at earlier levels. While in skills of social cognition (communication, social learning, theory-of-mind), children already out-performed *Pan* apes at 2 years, and increased the discrepancy even more by 4 years. They documented an emergence of goal understanding and of intention emulation at 2 years of age in humans and at 7 years or more in *Pan* apes. However, results comparing children and apes should be viewed with caution because of anthropocentric interpretative bias, inadequate controls and lack of ecological validity (Leavens et al., 2017). Nevertheless, this may indicate that the development of physical and basic-goal stances had different trajectories in humans *versus Pan* apes after separation from the common ancestor.

### Evolutionary Psychology

Teleology “arguably constitute[s] an evolved mode of interpretation built into the human mind” (Tooby and Cosmides, 2016, p. 14). Barrett (2015) stated that mindreading “has all the hallmarks of a complexly organized adaptive system: it likely evolved in steps rather than all at once, and it likely involves the interplay of multiple, specialized mechanisms” (p. 129). Indeed, as shown above, belief-stance possesses many properties of psychological adaptations: special design, underlying genetic variation, neurochemical specialization, cognitive modular integration, high efficiency/intricacy, functionality, developmental and phylogenetic dissociation from other domains, universal ontogenetic trajectory, cross-cultural universality. Given all the costs and drastic effects of minimal social interaction upon autistic individuals lacking mindreading, belief-stance also has benefits as a social instinct. Possible evolved functions of ToM are intentional communication, repairing communication, teaching others, persuasion, deception, devising shared plans and goals, sharing a focus or topic of attention, and pretending (Baron-Cohen, 1999; Brüne and Brüne-Cohrs, 2006). Smith (2006) argues that ancestral ToM enhanced social functioning and behavior prediction, and it facilitated conversation, social expertise, parental care, and deception. Thus, by improving detection, understanding, and forecasting of adult human behavior, the belief stance might have improved survival and reproduction (i.e., fitness). All of those possible ancestral adaptive values of ToM should be tested properly to qualify as truly adaptive advantages (Schmitt and Pilcher, 2004). Brüne and Brüne-Cohrs (2006) traced back the phylogeny of ToM and argued that it evolved from the capacity to monitor biological motion and from imitation behavior. Barrett et al. (2005) found cross-culturally that intention can be accurately perceived from visual motion cues alone.

Although less-studied, the physical, design, and basic-goal stances also provide evidence of special design, neurochemical specialization, cognitive modular integration, high efficiency/intricacy, functional, developmental, and phylogenetic dissociation from other domains, specific ontogenetic trajectory, and even older phylogenetic roots. All the available evidence supporting the distinction among the four stances affirms that they are specialized for tracking different, recurrent, and evolutionary relevant phenomena. The physical stance may have helped survival by improving understanding, forecasting, and coping with the physical world. The design stance may have promoted survival and reproduction by improving detection, use, and creation of functionality. The basic-goal stance may have benefited survival by improving detection, understanding, and forecasting of agents, particularly non-human prey and predators. Indeed, Csibra and Gergely (2007) argued that goal-directed reasoning promotes on-line prediction and social learning by drawing action-to-goal and goal-to-action inferences.

The fact that in nature time, energy, and resources are limited and that individuals compete is related to the evolution of the common underlying presumption of rationality/optimization (Dennett, 1989; Gergely and Csibra, 2003; Hudson et al., 2018) among the three truly teleological stances. In the face of limiting resources and competition, natural selection influences the evolution of fairly well-designed and roughly optimized body parts and behavioral strategies (Dennett, 1995; Ayala, 2016; Tooby and Cosmides, 2016), which have co-evolved with the perceptual and inferential abilities of design, basic-goal and belief stances (Hudson et al., 2018). Economy, efficiency, and functionality are among the hallmarks for identifying adaptations (Buss et al., 1998; Schmitt and Pilcher, 2004). Moreover, the optimal foraging theory explains the presumption of optimized choice for food in many species (Pyke et al., 1977). Hence, it makes sense that design, basic-goal and belief stances assume and yield rationality/optimality from body parts, behavioral strategies, and psychological tactics (cf., Schoemaker, 1991).

Importantly, there is a strong evolutionary reason for the adaptiveness of anthropomorphic tendencies. Rather than being a simple byproduct or another flaw in human cognition, propensity toward anthropomorphisms may be an evolved design feature. Guthrie (1993), Atran and Norenzayan (2004), Broaddus, unpublished, Beck and Forstmeier (2007), Shermer (2008, 2011), and Engvild (2015) explained the propensity to over-attribution using the ‘better easily triggered than sorry’ logic of Error Management Theory (Haselton and Buss, 2000): Because the costs of false-negatives in ancestral environments were much higher than those of false-positives, the underlying psychological mechanisms were selected to be biased toward the least costly mistake, hence false-positives abound. Not detecting harmful properties of parts of plants/animals or hidden traps on the way (design stance), of harmful movements of predators (basic-goal stance), or of an ambush and humans with harmful/cheating first or second intentions (belief stance) could be lethal. In contrast, over-detecting harmful functions, goals, or planned intentions where there were none would hardly be lethal. When we feel fear, many internal reactions occur, one of which is that signal detection thresholds shift. Less evidence is needed to trigger the threat response, thus more valid positives will be perceived at the low cost of a higher rate of false alarms (Tooby and Cosmides, 2008). Foster and Kokko (2009) tested this logic using evolutionary modeling and concluded that natural selection favors strategies that make many incorrect causal associations in order to establish those that are essential for survival and reproduction. Similarly, Brown et al. (1999) modeled optimality in prey-predator systems and found that one endpoint on an ecology of fear continuum favors the evolution of prey becoming more vigilant or moving away from *suspected* predators. Therefore, natural selection has made us more teleologically apprehensive and vigilant. This line of evolutionary reasoning can explain for instance why there is a brain component (lateral prefrontal cortex and dopaminergic system) devoted to the execution/application of mental states, why paranoia (negative intentions) is more common than erotomania, why people anthropomorphize more when alone or afraid, and why the striatum related to ToM is also activated by aversive/intense or novel/unexpected stimuli. Still, evolution is more than natural selection, thus other evolutionary factors may also play a role.

This section presented the main genetic, neural, cognitive, psychiatric, developmental, comparative and evolutionary/adaptive evidence pointing to the existence of the four distinct stances (physical, design, basic-goal, belief). Following Schmitt and Pilcher’s (2004) framework for integrating evidence of adaptation, I have presented a comprehensive cross-disciplinary integration of results supporting the plural nature of teleological reasoning mechanisms. It also demonstrates that overly active calibration is possibly an evolved design feature to avoid harmful contexts that explains the widespread occurrence of anthropomorphisms. This confirms and expands the depth of the presumed biopsychological roots of mental anthropomorphism, and sets the stage for exploring the occurrence of anthropomorphism in philosophy of biology and teaching of evolution with the mosaic of three overactive psychological tendencies in mind.

## Reuse of the Three Anthropomorphic Tendencies in Understanding Life and Evolution

As part of our evolved intuitive/folk: physics, engineering/morphology/social contribution, behavior-reading and psychology/ethics; the four stances (physical, design, basic-goal, belief) inescapably get engaged while reasoning about modern science due to input similarities between the studied objects/processes and the evolved proper domains. In connection with other tendencies, those four systems exert a considerable influence on science matters, mostly on biology, but also on chemistry and physics (Kampourakis, 2007). This does not mean that biological science is less scientific, not objective neither that it cannot be materialistically explained (Mayr, 2004). Particularly, design, basic-goal, and belief stances are commonly related to the comprehension of processes and products of evolution by natural selection with negative and positive consequences.

On the positive side, they enable specialized scientific thinking by providing its cognitive foundations (e.g., inference, motivation, affinity) upon which academic competency is built (Geary, 2002). Moreover, relying on the three genuinely teleological stances while reasoning about biology and evolution leads to the pragmatic advantage of engaging a high-powered, acute, and skillful use of our minds; they easily organize data, explain interrelations, and integrate disparate topics (Dennett, 1989, 1995; Pinker, 2007; Haig, 2012). Lombrozo and Gwynne (2014) found that compared with a mechanistic mode of explanation (physical stance), properties of species and artifacts that are explained functionally (design stance) are more likely to be generalized on the basis of shared functions. Hence, they also promote generalization.

Furthermore, the heuristic value in terms of fostering new research questions and discoveries when asking for reasons, roles, goals, strategies, and values using “why?” and “what for?” questions is also crucial and documented (Schaffner, 1993; Buss et al., 1998; Dennett, 1989, 1995; Panksepp, 2003; Mayr, 2004; Haig, 2012; Tooby and Cosmides, 2016). Consequent metaphorical thinking helps researchers to model some processes/behaviors and use the grammatical construction of the active voice to didactically explain the dynamics to others (Ridley, 2003; Blancke et al., 2014; Galli, 2016). Even Darwin (1861) noted that ‘natural selection’ literally is a misnomer that implies the active power of a personified nature, but he argued that such metaphorical expressions are also found in chemistry and physics and added that they are important and almost necessary for brevity.

However, on the negative side, they can be involved in at least 8 classes of problems/controversies (cf., Mayr, 2004):

### Over-Activation-Without-Over-extension Type of Anthropomorphism

This occurs when there is over-attribution within the appropriate domain. For instance, attributing functional design to all aspects of a designed system, e.g., pan-adaptationism (Varella et al., 2013); attributing internal desire/need to all agent actions, e.g., fundamental attribution error (Granot and Balcetis, 2014); attributing intentional belief to all human actions, e.g., teleological obsession/over-active intentionality bias (e.g., Rosset, 2008).

### Over-Activation-With-Over-Extension Type of Anthropomorphism

This occurs when over-activation is directed to an inappropriate domain. Based on **Table 1**, one can try to explain all four groups of material phenomena with all four modes of thought and observe the types of anthropomorphic extrapolations whereby schema from a given stance are erroneously transferred to an unsuitable phenomenon. **Table 2** explores this insight by presenting all specific answers to a ‘why’ question through mapping the non-mutually exclusive proper and improper use of each stance.

**Table 2:** Possible answers to a ‘why’ question about the behavior of four typical cases of material phenomena using all four modes/stances of thought, and its relation to kinds of anthropomorphic errors.

Why does...	(1) Physical stance	(2) Design stance	(3) Basic-goal stance	(4) Belief stance
(IV) The woman speak?	Because she produces patterned sound waves	She is naturally designed to speak to better communicate	She just desires to speak now	She knows why she intends to speak about that now
(III) The ant walks?	Because of coordinated leg movements	It is naturally designed to walk to help foraging locomotion	It needs to follow the trail	It knows why it intends to seek food
(II) The heart beat?	Because of rhythmic contractions	It is naturally designed to pump to circulate the blood	It wants to pump	It knows why it is important to keep pumping
(I) The continent move?	Because of cyclical mantle convections	It is programmed to move to help speciation	It feels like moving	It knows why it should move

		Gray area show corresponding over-activation-with-over-extension anthropomorphisms.		

Because the mechanistic/physical stance is non-teleological and answers a ‘why’ question as a ‘how come’ question, instead of ‘what for’ (Dennett, 2017), it never generates mental anthropomorphism. The design/functional stance generates anthropomorphism only when applied to non-designed physical phenomena (cell 2-I in the **Tables 2**, **3**), e.g., function compulsion (Kelemen and Rosset, 2009). Not surprisingly, most anthropomorphisms come from the cognitive devices focused on agent interpretation. The basic-goal stance generates anthropomorphism when used to explain non-agent systems (cells 3-I, 3-II). The intentional/belief stance may generate over-extended anthropomorphism when applied to all other domains (cells 4-I, 4-II, 4-III). By far the higher-order belief stance generates the majority of anthropomorphic acts given its narrow focus and high activity in a socially complex species such as *Homo sapiens* (cf., Wilson, 2012). Thus, the design, basic-goal and belief stances generate at least six different predictable acts of mental anthropomorphism. In **Table 3** I try to specify each occurrence of overextended type of mental anthropomorphism. **Tables 2**, **3** are important because, as Dacey (2017) argued, warning against ‘anthropomorphism’ in general is too vague to be helpful, thus the more we can identify specific errors, the better positioned we are to increase awareness of their occurrence and underlying causes, in order to avoid them. Dacey (2017) mentioned several variants of anthropomorphisms within the field of animal behavior.

**Table 3:** Specific label to each over-activation-with-over-extension type of Anthropomorphic error.

Mental stance in use	Type of phenomena focused	Type of error incurred
Design/functional stance	Physical phenomena	Promiscuous teleology Pan-function compulsion
Basic-goal stance	Physical phenomena	Pan-agenticity
	Designed mechanism	Object Agenticity
Belief stance	Physical phenomena	Pan-psychism
	Designed mechanism	Object Psychism
	Animal behavior	Animal Psychism

The three teleological stances also may be extrapolated to other phenomena, fictional or non-fictional. Basic-goal and belief stances can animate fictional agents such religious, mythological, folkloric and extraterrestrial ones (Guthrie, 1993; Shermer, 2011; Blancke and De Smedt, 2013). The non-fictional phenomena that the human mind surely was not evolved to grasp and which involve basic-goal and belief stances include: the dynamic of the market economy, e.g., the invisible hand (appearance of intentional design in large-scale results of human unintended consequences of collective action), and natural selection (appearance of intentional choice in populational results of non-random differences in reproduction). Those two phenomena share some conceptual connection (Carey, 1998). Although metaphorically anthropomorphic, the use helps to better grasp these abstract population dynamic (Darwin, 1861; Dennett, 1995, 2017; Pinker, 2007; Blancke et al., 2014). However, the general hyper-active influence of basic-goal and belief stances on understanding natural selection is called “Darwinian paranoia,” that is the propensity to think of all evolutionary outcomes in terms of an agent’s reasons, plots, and strategies (Francis, 2004; Godfrey-Smith, 2009).

Interestingly, the distinction among design, basic-goal, and belief stances helps to explain specificities and to organize a variety of misunderstandings regarding selectionism and adaptationism. By stressing the centrality of function, the design stance may be mainly responsible for “naïve adaptationist” (i.e., conviction that function is the only explanation for why traits evolve) described by Kelemen (2012), and “if a trait is not an adaptation, it is not evolved” (Varella et al., 2013). By stressing need, attempt, and goal respectively, the basic-goal stance might be the main reason for mistaken explanations described by Kelemen (2012), such as “basic need-based” (e.g., giraffes got long necks because they needed them to reach high food), and “elaborated effort need-based” (e.g., giraffes got long necks through repeatedly trying to eat highly positioned leaves or fruit on trees) and “basic function-based” (e.g., “giraffes got long necks so that they can reach high food”). By stressing premeditated precise adjustments, the belief stance may be the main reason for mistaken “elaborated design need-based” explanations (e.g., giraffes got long necks because Nature transformed them so that they could reach food at the tops of trees, in order to survive) described by Kelemen (2012). In a systematic review, Varella et al. (2013) compiled 22 misunderstandings in applying evolution to human mind and behavior; two of them involve the conflation of basic-goal and design stances into belief stance. On “intentional maximization of fitness,” the evolutionary gene’s point of view (heuristic over-extension of the basic-goal stance) is equated to human personal intention. On “confusion between individual intention and adaptation’s design,” the functional design of mental adaptations is equated to personal intentions.

Gregory (2009) reviewed studies on the quality of understanding about natural selection and found that the reliance on ‘need’ appears in mistakes about the origin of new traits, inheritance, and adaptation. Of 42 studies he reviewed, at least 13 found mistakes attributing evolutionary/adaptive change in response to need, 11 found use and disuse, 6 found mistakes were related to want/intent, 4 to teleology, 3 to anthropomorphism, 2 to goal-directness, 2 to directed mutation. All of these mistakes mostly were influenced by basic-goal and belief stances. They combined incorrect underlying premises about mechanisms and deep-seated cognitive biases (Gregory, 2009). These findings indicate that important causes of widespread misunderstanding about natural selection are cognitive/psychological (Kelemen, 2012; Varella et al., 2013; Blancke et al., 2014). They are much deeper than lack of acceptance, media exposure, lack of formal education, or religious impediment (cf., Rosengren et al., 2012).

### Interaction With Other Psychological Tendencies Such as Perfectionism, Anthropocentrism, and Internal/External Distinction to Generate More Misunderstandings

Varella (2016) showed that different intuitive concepts such as fixism, essentialism, perfectionism and anthropocentrism easily could amalgamate with pan-adaptationism, and with each other, to form hybrid misunderstandings with a strong intuitive appeal. The conjunction of anthropocentrism and the design stance give rise to the common notion that ‘humans exist in order to be the apex of evolutionary tree’ (e.g., Sandvik, 2008) and that ‘everything in nature is made to serve humans.’ The mixture of pan-adaptionism, perfectionism and transformationism originates in mistaken ‘cosmic teleology’ (i.e., tendency toward progress and to ever-greater perfection; Mayr, 2004) and ‘evolution as perfectionist’ (Varella et al., 2013). Although fixist, Aristotle’s original conception of teleology in nature (i.e., ‘nature does nothing in vain’) is a mixture of pan-adaptionism and perfectionism (Varella, 2016). It survived 24 centuries throughout history to be dismantled only by Darwin using non-teleological terms, such as randomness and genealogical inertia: the stamp of inutility (Solinas, 2015). However, the fact that Darwin discredited both creationist and Aristotelian teleology does not mean that he totally extinguished teleology in biology (Mayr, 2004; Ayala, 2016). “The irony is that Darwin’s discovery of natural selection did not obviate seemingly “teleological” concepts; it legitimized them, by showing how and why the consequences of biological phenomena constitute an essential part of the explanation for their existence” (Daly and Wilson, 1995, p. 35).

Another amalgamation occurs between teleological reasoning and the intuitive internal/external distinction of causal factors. Gregory (2009) characterized anthropomorphic misconceptions as either internal (i.e., attributing adaptive change to the intentional actions of organisms) or external (i.e., conceiving of natural selection or “Nature” as a conscious agent. Likewise, Godfrey-Smith (2009) distinguished two explanatory schemata when anthropomorphizing nature: The paternalist [perfectionist] schema, a benevolent agent who intends that all is ultimately for the best, and the paranoid schema, a hidden collection of agents pursuing agendas that impede our human interests. Regarding internal amalgamation, it also may intermix with essentialism to originate “Adaptation equals gene” (e.g., gene for aggression) and “selfish gene equals selfish person” (Varella et al., 2013). Moreover, essentialism alone induces the focus on the individual rather than the population (e.g., individual organism changing/evolving; Gregory, 2009), so that it increases the odds of the basic-goal stance providing need-based explanations. This indicates that common Lamarckian mistaken interpretations about ‘need’ and ‘trying’ occur because students [and Lamarck] share the same intuitive bias rather than students being directly and deeply influenced by his theorizing. Kampourakis (2013) warned against the use of the label “Lamarckian” to inappropriately mask the variety of teleological explanations that students give, also because technically speaking, most of their explanations are not actually Lamarckian (Kampourakis and Zogza, 2007).

### Intuitive Folk Dynamics at Odds With Current Scientific Attitudes

Some authors claim that teleological explanations are more appealing and preferable to causal/mechanistic ones and would steer people away from ‘how’ questions, causal/physical modes of explanations, or empirical testing. Godfrey-Smith (2009) claimed that once teleological modes of thinking are turned on, they are difficult to abandon, because they have a compelling, addictive, and narrative appeal, and after starting to understand a phenomenon in terms of a persuasive rationale, people become reluctant to settle for less. The appealing narratives of agent stances may make people, including some scientists, readily satisfied with “just-so stories,” but empirical verification should always be the gold-standard, even within the exaptationism program (Andrews et al., 2002). However, this problem should not be considered automatically as an inherent aspect of expert evolutionary reasoning in general (Varella et al., 2013). Godfrey-Smith (2009) also asserts that explaining life in terms of agents’ agenda “makes sense” in a way that efficient causes cannot. Children possess a generalized bias in favor of teleological or purpose based explanations (Kelemen, 2012). Adults with poor inhibitory control in time-constrained contexts tend to broadly explain living and non-living natural phenomena by reference to a purpose (Kelemen and Rosset, 2009). Even physical scientists and humanities scholars accepted more unwarranted teleological explanations when working at speed, despite maintaining high accuracy on control items (Kelemen et al., 2013). However, Lombrozo and Carey (2006) showed that in less-constrained situations, teleological explanations are not easily accepted; only when the function invoked in the explanation conforms to a predictable pattern and when the function played a causal role in bringing about what is being explained. Heussen (2010) showed that when subjects focused on properties of body parts, causal and functional explanations were viewed as equally plausible, while for artifacts, causal explanations even were preferred over functional explanations. Richardson (1990) found that students tend to prefer teleological explanations 61% of the times over mechanistic explanations for body function, but after a short-term lecture with discussion regarding teleological and mechanistic thinking the preferences for teleological explanations were 12%. Thus, although there is a default bias toward purposeful explanation, there is also room for controlled, secondary modulation and inhibition through learning (e.g., Friedler et al., 1993). Moreover, Zohar and Ginossar (1998) showed that the acceptance of anthropomorphic or teleological formulations by high school students does not necessarily imply anthropomorphic or teleological reasoning, and the use of a textbook with numerous teleological/anthropomorphic formulations by biology students is not followed by an increase in students’ application of teleological/anthropomorphic explanations.

### Conceiving All Versions of Anthropomorphism and Teleological Reasoning as Stemming Only From the Belief Stance/Folk Psychology

The lack of a clear distinction among the three cognitive systems of teleological reasoning (design, basic-goal, and belief stances) has led authors from one side to generalize it as a simply metaphorical but not a real explanation. The other side keep an overly suspicious view about any ‘in order to’ type of argument, denying it completely or even questioning the sanity of biologists. For instance, Mahner and Bunge (1997) clearly stated that in Biology,

“we meet an almost schizophrenic situation. On the one band, many authors maintain that teleological concepts are legitimate in biology or are even constitutive of biology’s (alleged) autonomy; on the other hand, they take pains to point out that biological teleology is somehow not a genuine teleology, but only an as-if-teleology, occasionally called ‘teleonomy.’ A similar contradiction can be found in the assurance that teleological explanations in biology could be translated into non-teleological ones, but eliminating teleology altogether would be impossible because “something would get lost” by doing so. Thus, biologists apparently cannot live with teleology but they cannot live without it either” (p. 367).

In the same vein J. B. S. Haldane famously said that teleology is like a mistress to a biologist because he cannot live without her, but he is not willing to be seen with her in public.

I argue that it is not the case anymore. Contemporary biologists do not need to hide their teleological proclivities nor disguise them as ‘as-if-teleology.’ Considering distinctions displayed in **Table 1** and Section “Proximate and Distal Evolutionary Facets Underpinning Mental Anthropomorphisms”, it is clear that both the phenomena explained teleologically and the cognitive mechanisms used thereof are heterogeneous. Thus, beside the misattributions, there are plenty of genuine, legitimate, and literal uses of teleological explanations about functions, animal needs, goals, intentions, and lots of heuristic metaphorical uses of teleological clauses about natural selection, selective pressures, evolved strategies, and a gene’s eye view that do not necessarily engage the premeditated belief stance, hence, strictly speaking they are not an *anthro*pomorphic mistakes. The fact the one can mistakenly interpret those same explanations as over-extending the belief stance (**Table 2**) does not mean that it is all that is.

When considering the belief stance as the only genuine teleology, and thus inappropriate for biology, one embraces an outdated high level of anthropocentrism that hinders nuanced multifaceted approaches. We now know that other animals also have needs, desires, goals and intentions (Allen and Bekoff, 1999; Panksepp and Biven, 2012) and that they perceive, attribute and process basic goals in conspecifics and other species (Emery and Clayton, 2009; Hudson et al., 2018). Thus, talking about the needs, goals and intentions of other primates or mammals is technically not *anthro*pomorphism (cf., Mitchell and Hamm, 1997). Furthermore, because tool use/manufacture appears across three phyla and seven classes of animals, with Passeriformes and Primates presenting diverse uses (Bentley-Condit and Smith, 2010; Shumaker et al., 2011), not even the classical ‘watchmaker’ type of designer analogical explanation should be considered *anthro*pomorphism anymore. Interestingly, although the basic-goal stance has evolved mostly to focus on animals, newer research indicates that plants sense, process experiences, memorize, learn, communicate and show adaptive behavior (Baluška and Mancuso, 2007). Thus, the new field of plant neurobiology (Brenner et al., 2006; Calvo, 2016) already is recruiting the basic-goal stance to heuristically interpret these findings, which exasperates critics, but again it is technically not *anthro*pomorphism. Overall, this is a promising case in which new convergent evidence from bio-psychology can help bio-philosophers (cf., Livingstone, 2017) to make updated conceptual distinctions clarifying new avenues of enquiry.

### Gateway to Mystic, Religious, and Conspiratorial Reasoning That Poses an Obstacle to Science

Only children show “promiscuous theism” (Kelemen, 2004), but Kelemen and Rosset (2009) found no link in adults between belief in God and acceptance of unwarranted teleological ideas. Lombrozo et al. (2007) found that patients with Alzheimer’s disease have a robust preference for teleological explanations without the promiscuous theism, which indicates that promiscuous teleology is not a consequence of believing that everything was designed by a divinity nor leads toward it. The opposite seems to be the case, since the reduced belief in a God on autistic patients and in men is mediated by their lower mentalizing capacities (Norenzayan et al., 2012).

### Prohibition of All Teleology in Science

Although over-active teleological reasoning does not correlate or lead to religiosity, humans still have persistent teleological reasoning by default (Kelemen et al., 2013; Coley et al., 2017). Hence, in order to avoid the unwarranted forms of teleological anthropomorphism, science has become increasingly opposed to all types of teleology (cf., Nagel, 1961; Mahner and Bunge, 1997; Cummins and Roth, 2009), thus promoting anthropodenial (Mayr, 2004; Panksepp and Biven, 2012). However, once we realize that both the teleological phenomena and explanation are real and heterogeneous (**Table 1** and Section “Proximate and Distal Evolutionary Facets Underpinning Mental Anthropomorphisms”; cf., Dennett, 1989, 1995, 2017; Mayr, 2004), the possibility of ‘throwing the baby out with the bath water’ by prohibiting teleology becomes reality (cf., Zohar and Ginossar, 1998; Galli and Meinardi, 2011; Galli, 2016).

In general, for philosophy of science within the physicalist tradition, the anti-teleology movement means the correct rejection of animism, obscurantism as inherently non-scientific (e.g., Nagel, 1961), however, it may lead to issues of nomological reduction of all biological explanations, up to questioning the autonomy of biological sciences *per se* (Mayr, 2004; Ayala, 2016). In biology, anti-teleology means the correct rejection of vitalism (Mayr, 2004) leading to the precipitated rejection of the concept of a biological program (e.g., Mahner and Bunge, 1997). It also means the correct rejection of instructive models of adaptationism such as creationism, intelligent design, and Lamarckism (Cronin, 1993). But rejection may also lead to the long-standing dismissal of sexual selection and signaling evolution (Cronin, 1993; Miller, 2000), which are co-evolutionary processes guided by conspecifics although not fully thought-out by them. **Table 4** relates the contribution of each stance to the proper understanding of some evolutionary mechanisms. In psychology, the anti-teleology approach concerns the black box approach of early behaviorism and classical ethology by denying mentalistic terms, but also leads to denial of emotions, cognition, self-awareness and consciousness to other animals (Panksepp and Biven, 2012; Brejcha and Kleisner, 2016). According to Pinker (2007),

**Table 4:** Possible overlapping over-extended contributions of each of the four mental stances to correctly understand facets of some evolutionary mechanisms.

	Physical stance	Design stance	Basic-goal stance	Belief stance
Genetic variation	Randomly caused^∗^	Not directional	Not guided	Not premeditated
Natural selection	Non-randomly caused	Directional	Not guided	Not premeditated
Sexual/signaling selection	Non-randomly caused	Directional	Guided	Mostly not premeditated
Artificial selection	Non-randomly caused	Directional	Guided	Partially premeditated
Genetic engineering	Non-randomly caused	Directional	Guided	Highly premeditated

^∗^*Randomly caused in the sense that it does not co-vary with fitness, not in a sense that it cannot be non-randomly caused by specific mutagenic factors*.

“the biggest impediment to accepting the insights of evolutionary biology in understanding the human mind is in people’s tendency to confuse the various entities to which a given mentalistic explanation may be applied. (...) More generally, I think it was the ease of confusing one level of intelligence with another that led to the proscription of mentalistic terms in behaviorism and to the phobia of anthropomorphizing organisms or genes in biology. But as long as we are meticulous about keeping genes, organisms, and brains straight, there is no reason to avoid applying common explanatory mechanisms (such as goals and knowledge) if they promise insight and explanation” (p. 138–139).

### Backfiring Prohibition of Teleology

While speaking of purpose and design in nature seems to strengthen the creationists’ arguments, Dennett (2017) argues that to prohibiting all teleological reasoning as mere ‘jargon’ in biology can backfire badly. That is because by using the intuitive design stance anyone easily can find functions in the living world, and then conclude that biologists are reluctant to admit the manifest design because of the difficulty of explaining it without an intelligent designer. He suggests that instead of trying to convince lay-people that they do not really see the design they find in nature, we should rather try to persuade them that because of the cyclical, non-random and cumulative features of natural selection, there is real design in nature without a conscious premeditative all-knowing designer (Dennett, 2017).

This section explored the positive and negative aspects of the proper and overextended uses of the three teleological stances in relation to biological matters. I argued that recognizing the reality and distinctions among design, basic-goal, and belief stances is a key to illuminate and to better understand the logic underlying many of the issues involved: anthropomorphism, misunderstandings, seductive appeals, legitimacy controversy, gateway assumptions, prohibition, and its backfire effect. Importantly, the recognition of the multifaceted psychological nature of teleological reasoning enables new avenues for establishing a much more detailed taxonomy of anthropomorphisms (see **Tables 2**, **3**). The challenge now is how the recognition of the reality and distinction among design, basic-goal, and belief stances can help to alleviate most of the negative aspects.

## Workaround Educational Strategies

A better understanding of the three distinct deeply engrained neurocognitive teleological tendencies to anthropomorphize may help to illuminate ways of how to better deal, cultivate, canalize, and train them. Notably, their biological roots do not suggest that we should give up trying and embrace fatalism or naturalistic fallacy. As the biological nature of myopia did not deter the development of correcting glasses, the bio-psychological nature of teleological stances should assist us developing ‘trifocal glasses,’ in order to see clearly this tripartite distinction and to learn which one to use in which situation. Therefore, here I consider some strategies that are likely to succeed or fail in maximizing solutions to problems raised by anthropomorphism in philosophy and education.

One strategy likely to fail is the suppression of all teleological reasoning (Zohar and Ginossar, 1998; Galli and Meinardi, 2011; Galli, 2016). That is because, as for the prohibition of drugs (Levine, 2003), abstinence-only sex education (Stanger-Hall and Hall, 2011), or suppression of emotion during decision-making (Lerner et al., 2015), it always finds a clandestine route back. Their suppression is counter-productive, and they reappears devoid of regulation, with lower quality and with worse consequences. By suppressing teleological thinking in biology classes, one also restricts intuitive thinking mechanisms that would better suit the problem, so it is neither feasible nor advantageous to deter teleological thinking (Zohar and Ginossar, 1998; Galli and Meinardi, 2011; Galli, 2016). As with biology, mathematics is not fully intuitive to humans, but numeracy intuitions intrinsically available are not abandoned or suppressed just because they may lead to error or they are incongruent with current knowledge; instead they are rigorously trained, refined, and connected with other capacities and thinking strategies (Geary, 2002; Apperly and Butterfill, 2009). Ideally, this approach also should be developed for teleological thinking in biology.

Varella et al. (2013) highlighted some strategies to better deal with evolutionary misunderstandings in the classroom: Considering previous knowledge, emphasizing critical thinking, explicitly approaching mistaken explanations and their presumed implications, stressing the interference of evolved cognitive biases (e.g., essentialism and teleology), and using structured-active learning. Similarly, Nelson (2008) suggested directly address misconceptions and student resistance, focus on scientific and critical thinking, and use structured active learning extensively as effective strategies for teaching evolution. Nehm and Reilly (2007) found that active learning was more efficient than traditionally taught class in reducing occurrence of misconceptions (also called alternative conceptions) about natural selection. Richardson (1990) found that one short-term lecture explicitly distinguishing between teleological and mechanistic thinking when applied to body function was enough to keep preference for finalistic explanations over four-times lower than in control classes. Within a one-semester biology course, Stover and Mabry (2007) found improved student understanding of natural selection after they monitored teleological language, carefully dealt with misunderstandings, avoided using wrong teleological explanations, offered laboratory/problem-solving activities, and presented historical context. Global attempts in this direction, such as the Biology Critical Thinking Project, seem effective and promising (Zohar et al., 1994). The development of questionnaires and inventories such as the Conceptual Inventory of Natural Selection (Anderson et al., 2002; Nehm and Schonfeld, 2008) can also help instructors to test the effectiveness of their intervention. As a way to control implicit anthropomorphic biases, Dacey (2017) proposed a check-list including items that stress alternative hypotheses that might explain the behavior and items that systematically help to identify errors. The more detailed taxonomy of over-attributing anthropomorphisms, suggested by this present multifaceted approach, may help the development such a preventative checklist.

Dacey (2017) also suggests that ensuring that counter-stereotypical information is saliently available for reasoning is an efficient way to avoid intuitive anthropomorphism. This strategy is exactly what Darwin did by using randomness and genealogical thinking to break with the notion that everything in nature is perfectly adapted (Solinas, 2015). Indeed, Kampourakis and Zogza (2009) found that first teaching about fundamentals, biological organization, mechanisms of heredity, and the origin of genetic variation helped to overcome students’ preconceptions, and to achieve conceptual change. This change occurred because they put emphasis on the role of unpredictability and chance in the evolutionary process, which is incompatible with the idea of deliberated purpose/design in nature. Similarly, including historical processes (e.g., phylogenetic inertia) into the definition of adaptation may help students scrutinize intuitions about purpose and design in nature (Kampourakis, 2013). Within this strategy, educators should be aware that students may erroneously conclude that natural selection and everything in nature is random.

Many authors have explored non-suppressive teaching strategies aligned with the classical proposal to lift the taboos regarding teleology and anthropomorphism (Zohar and Ginossar, 1998). A specific strategy likely to succeed is to promote explicit control over the belief stance, circumscribing it and to decreasing its influence on the other stances’ domains, in order for them to work alone. Dawkins (1986) famously made the watchmaker blind as a way to stay with basic-goal teleological reasoning without the premeditative thought-out side of belief stance. Dennett (2013, 2017) argues that stressing the existence of ‘competence without comprehension’ is crucial for understanding how natural selection can promote efficient functional design but without reasoned planning. Blancke et al. (2014) argued that the natural-selection-as-metaphor-of-designer after being dissociated from its intentional overtones actually may aid an initially teleological need-based understanding of evolution, which consequently may function as a scaffold to build a more scientific understanding. Similarly, Galli (2016) emphasized the explicit analysis of the metaphor of design, in order to promote student’s meta-cognitive skills for recognizing, understanding, and regulating the metaphor of design in biology (cf., Galli and Meinardi, 2011). Legare et al. (2013) studied children’s understanding of evolutionary change by comparing the effectiveness of using desire-based/anthropomorphic narratives (intentional mental states) with need-based (no reference to desires or conscious intent from the organism) and natural selection language. They found that need-based and natural selection language had similar positive effects, while anthropomorphic mental languages was worse for facilitating accurate interpretation. The multifaceted nature of the teleological reasoning into design, basic-goal, and belief stances legitimizes this pedagogical approach.

Complementarily, this multifaceted approach suggests that strategies aiming to focus on natural selection in the non-living or non-human domains could be promising, given that distinct mental systems would be activated. Metaphorically referring to natural selection as a ‘goal-achieving system’ such as a filter, an organ like a simple kidney, a Genome Organizing Device (Ridley, 2003), a sorting algorithm (Dennett, 1995, 2013, 2017), a bottom-up crane instead of a top–down skyhook (Dennett, 1995, 2013) may aid in achieving a more accurate understanding, by getting a stronger mental grip from the design stance, while inhibiting conclusions based on pure chance or pure top–down deliberation/premeditation. In the same vein, approaching natural selection as an simple agent, such as a mindless *bricoleur* (tinkerer) (Jacob, 1977), or mother nature (Dennett, 1995, 2017) could help to better engage the basic-goal stance, again avoiding pure chance or pure premeditation kinds of reasoning.

Another strategy derived from the adaptive value of over-attribution tendencies would be to lower the level of anxiety/fear during teaching and examination about natural selection. Also given that hyper-mentality is directly related to dopamine levels, which is associated with enthusiasm and expectation (Shiota et al., 2017), preparing ‘super-engaging’ classes also may be contra-productive. Educators should never forget to address teleological and anthropomorphic misunderstandings together with other sources of bias such as essentialism, perfectionism and progressivism. Moreover, for every ‘why’ or ‘what for’ question answered, a corresponding ‘how’ question also should be addressed in order to give a more balanced view between causal and functional factors (cf., Hogan, 2017).

Furthermore, educational strategies should not ignore gender. This is because as we saw, on average, women more than men tend to over-attribute faces (Proverbio and Galli, 2016), have higher mentalizing (Warrier et al., 2017), and empathizing (Baron-Cohen, 2005; Varella et al., 2016). Thus, they might be more prone to anthropomorphic misunderstandings. In fact, Cunningham and Wescott (2009) found that females more than males tended to agree that species evolves ‘because individuals want to.’ The same way mentalizing partly explains the higher belief in a god by females (Norenzayan et al., 2012), it might also influence their lower focus on Science (cf., Jones et al., 2000; Sjøberg and Schreiner, 2010). By not capitalizing on mentalizing, anti-teleological educational approaches might thus hinder female intuitive comprehension of biosciences. In order to better-tailor educational strategies that do not obstruct women’s interest in science, future studies should control for sex, gender and cognitive style of the participants.

Future research should thoroughly test and replicate all those propositions and pin down the internal and external modulators of each over-estimating tendency, in context, in order to foster the development of better intervention strategies.

## Conclusion

In this review, I have presented a promising multifaceted approach to advance the debate regarding the psychological underpinnings of anthropomorphisms, to further support the materialistic and qualified lifting of the taboos regarding teleology and anthropomorphism in biology, philosophy and education, and to improve on pedagogical strategies aiming on maximize its positive sides and minimizing its negative aspects.

I firstly compiled and integrated 13 conceptual distinctions of folk finalistic reasoning into four psychological inference systems (physical, design, basic-goal, and belief stances), with the latter three being truly teleological, and thus prone to anthropomorphisms. I then integrated the cross-disciplinary genetic, neural, cognitive, psychiatric, developmental, comparative, and evolutionary/adaptive evidence that converges to support the existence of the four distinct stances. This exercise also revealed that the over-reactive calibration of the three teleological systems, which makes them more prone to anthropomorphisms, is possible an evolved design feature to avoid harmful contexts. This effort has confirmed and expanded the depth of the bio-psychological roots of mental anthropomorphism which indicates the unfeasibility of totally suppressing them.

Due to over-activation and input similarities between the studied objects/processes and the focused domain of each of the four stances (physical, design, basic-goal, belief), they inevitably get engaged while reasoning about modern science. Design, basic-goal, and belief stances have much to offer to biology: they provide cognitive foundations, express a high-powered explanatory system, promote functional generalization, foster new research questions and discoveries, enable metaphorical/analogical thinking, and didactically explain with brevity. This impressive positive side suggests that it can be valuable to find better ways to engage with their problematic sides and so legitimize responsible use. I showed that recognizing the reality and distinctions among design, basic-goal, and belief stances elucidates much of the logic underlying many of the issues/problems involved: Types of anthropomorphism, variety of misunderstandings, its seductive appeal, legitimacy controversy, gateway assumptions, prohibition and its backfire effects. Additionally, this multifaceted approach opens new avenues for establishing a much more detailed taxonomy of over-attributing anthropomorphisms, including distinctions such as bodily *versus* mental, legitimate *versus* illegitimate, with *versus* without over-extension, extended over each other stances’ proper domains (**Table 3**) *versus* over new topics, fictional *versus* non-fictional new phenomena, pure *versus* combined with other biases, didactically promising *versus* problematic. Paraphrasing Pinker (2007), as long as we are meticulous about keeping straight design stance, basic-goal stance and belief stance, there is no reason to avoid carefully applying teleological reasoning to biology.

This line of reasoning stressing multifaceted stances is important because that it also offers a psychological substrate that is empirically based for anchoring definitions and terminology. Given that mental anthropomorphisms are addressed in different fields there is much variation in arbitrarily subjective definitions and inferences. Thus, an objective interdisciplinary approach grounded in the three teleological stances may make cross-fields discussions more profitable. Similarly, future experiments in education where researchers present teleological statements for students to judge should more precisely circumscribe each stance.

I hope this review usefully brings together related disparate academic literature in a way that offers the elements for fostering interdisciplinary discussion and research toward a more refined and bio-psychologically based way of thinking about anthropomorphism and teleology.

## Author Contributions

MV conceived, researched, organized, wrote, corrected, and formatted the entire manuscript.

## Conflict of Interest Statement

The author declares that the research was conducted in the absence of any commercial or financial relationships that could be construed as a potential conflict of interest.

## Footnotes

1 Richard Dawkins at the 20 min 12 s of “The Big Question: Why Are We Here?” https://www.youtube.com/watch?v=sWaLBiM6O5k
